# Inhibition of the group I mGluRs reduces acute brain damage and improves long-term histological outcomes after photothrombosis-induced ischaemia

**DOI:** 10.1042/AN20130002

**Published:** 2013-07-11

**Authors:** Hailong Li, Nannan Zhang, Grace Sun, Shinghua Ding

**Affiliations:** *Dalton Cardiovascular Research Center, University of Missouri-Columbia, MO 65211, U.S.A.; †Department of Biological Engineering, University of Missouri-Columbia, MO 65211, U.S.A.; ‡Department of Biochemistry, University of Missouri-Columbia, MO 65211, U.S.A.

**Keywords:** apoptosis, calpain, cell proliferation, glial scar, mGluRs, neuronal death, penumbra, photothrombosis, tissue loss, AMPAR, α-amino-3-hydroxy-5-methyl-4-isoxazolepropionic acid receptor, BrdU, bromodeoxyuridine, CHPG, (RS)-2-chloro-5-hydroxyphenylglycine, CNS, central nervous system, DG, dentate gyrus, DHPG, (RS)3,5-dihydroxyphenylglyine, FJB, fluoro-Jade B, GCL, granular cell layer, GFAP, glial fibrillary acidic protein, GluR, glutamate receptor, GPCR, G-protein coupled receptors, mAb, monoclonal antibody, MCAo, middle cerebral artery occlusion, mGluR, metabotropic glutamate receptor, MPEP, 2-methyl-6-(phenylethynyl)-pyridine, NIH, National Institutes of Health, NMDAR, *N*-methyl-D-aspartate receptor, PFA, paraformaldehyde, PLC, phospholipase C, PT, photothrombosis, RB, rose Bengal, SGZ, subgranular zone, TBST, Tris-buffered saline containing 0.1% (v/v) Tween 20, TUNEL, terminal deoxynucleotidyltransferase-mediated dUTP nick-end labelling

## Abstract

Group I mGluRs (metabotropic glutamate receptors), including mGluR1 and mGluR5, are GPCRs (G-protein coupled receptors) and play important roles in physiology and pathology. Studies on their role in cerebral ischaemia have provided controversial results. In this study, we used a PT (photothrombosis)-induced ischaemia model to investigate whether antagonists to the group I mGluRs may offer acute and long-term protective effects in adult mice. Our results demonstrated that administration with mGluR5 antagonist MPEP [2-methyl-6-(phenylethynyl)-pyridine] or mGluR1 antagonist LY367385 by intraperitoneal injection at 3 h after PT decreased brain infarct volume evaluated one day after ischaemia. Additive effects on infarct volume were observed upon co-injection with MPEP and LY367385. These antagonists also significantly alleviated neurodegeneration and apoptosis in the penumbra. In addition, when evaluated 2 weeks after PT, they reduced infarct volume and tissue loss, attenuated glial scar formation, and inhibited cell proliferation in the penumbra. Importantly, co-injection with MPEP and LY367385 reduced the expression levels of calpain, a Ca^2+^-activated protease known to mediate ischaemia-induced neuronal death. Injection of calpeptin, a calpain inhibitor, could inhibit neuronal death and brain damage after PT but injection of calpeptin together with MPEP and LY367385 did not further improve the protective effects mediated by MPEP and LY367385. These results suggest that inhibition of group I mGluRs is sufficient to protect ischaemic damage through the calpain pathway. Taken together, our results demonstrate that inhibition of group I mGluRs can mitigate PT-induced brain damage through attenuating the effects of calpain, and improve long-term histological outcomes.

## INTRODUCTION

Glutamate, the principal excitatory neurotransmitter in the CNS (central nervous system), not only can open ionotropic GluRs (glutamate receptors), such as NMDARs (*N*-methyl-D-aspartate receptors) and AMPARs (α-amino-3-hydroxy-5-methyl-4-isoxazolepropionic acid receptors), but also can activate mGluRs (metabotropic GluRs). mGluRs are GPCRs (G-protein-coupled receptors) primarily localized in the CNS. Based on the sequence homology, signal transduction pathways, and pharmacological sensitivities, mGluRs are classified into three groups, i.e. group I, II and III mGluRs (Pin and Duvoisin, 1995; Schoepp et al., 1999; Niswender and Conn, 2010). The group I mGluRs, including mGluR1 and mGluR5, are expressed in neurons as well as in astrocytes. They are coupled to *G*_q_/*G*_11_, and can be activated by DHPG [(RS)3,5-dihydroxyphenylglyine]. Stimulation of mGluR1 and mGluR5 is coupled to activation of PLC (phospholipase C), which leads to the liberation of IP_3_ and subsequently release of Ca^2+^ from the ER (endoplasmic reticulum) through activation of IP_3_ receptors. In neurons, mGluR1 and mGluR5 are localized in the postsynaptic density area and their activation can modulate synaptic plasticity and neuronal excitation (Niswender and Conn, 2010). Astrocytes also express group I mGluRs (Zonta et al., 2003; Gwak and Hulsebosch, 2005; Wang et al., 2006; D’Antoni et al., 2008) and their presence has been suggested to play multiple physiological roles in CNS through Ca^2+^ signalling. Astrocytes can respond with Ca^2+^ increase to synaptically released glutamate (Porter and McCarthy, 1996; Pasti et al., 1997). On the other hand, stimulation of group I mGluRs in astrocytes with agonist DHPG can induce the release of glutamate through a Ca^2+^-dependent signalling pathway, and in turn, modulates neuronal excitability through activation of the NR2B-containing NMDARs (Fellin et al., 2004; Ding et al., 2007).

In pathological conditions, group I mGluRs are promising therapeutic targets in acute and chronic injury and neurodegeneration. mGluR1 antagonists were shown to attenuate neuronal death after mechanical injury *in vitro* and brain trauma *in vivo* (Faden et al., 2001). The mGluR5 antagonist MPEP [2-methyl-6-(phenylethynyl)-pyridine] could also alleviate NMDA-induced neuronal death (O’Leary et al., 2000). However, the role of group I mGluRs in animal models of ischaemia, remains controversial and the long-term effects of their antagonists on stroke outcomes have not been well investigated in details. An early study showed that knockout of mGluR1 in mice did not exhibit the neuroprotective effect (Ferraguti et al., 1997). On the other hand, in a rat model of focal cerebral ischaemia induced by MCAo (middle cerebral artery occlusion), administration of mGluR1 antagonist LY367385 immediately after ischaemia appeared to show neuroprotective effects (Kohara et al., 2008; Murotomi et al., 2008, 2010). Infusion of another mGluR1 antagonist YM-202074 for up to 24 h after MCAo also produced neuroprotective effect when evaluated 7 days later (Kohara et al., 2008). It was reported that mGluR1 agonist EMQMCM was neuroprotective, whereas mGluR5 antagonist MPEP was not neuroprotective in neonatal rats using the HI (hypoxia-ischaemia) model. On the other hand, MPEP was neuroprotective in the gerbil model of forebrain ischaemia (Makarewicz et al., 2006). In the rat MCAo model, it appeared that administration of both EMQMCM and MPEP were protective although their long-term effect was not assessed (Szydlowska et al., 2007). It is intriguing that both antagonist MPEP and agonist CHPG [(RS)-2-chloro-5-hydroxyphenylglycine] of mGluR5 have neuroprotective effects in rat MCAo model (Bao et al., 2001), whereas CHPG has no effect on brain injury in the endothelian-1-induced focal ischaemia model (Riek-Burchardt et al., 2007). These conflicting results on the role of these antagonists in ischaemia might have resulted from the use of different animal species, different ischaemia models and different developmental stages of animals.

In the present study, we investigated the role of mGluR1 and mGluR5 in neuronal damage in adult mice using the PT (photothrombosis)-induced ischaemia model established in our laboratory (Ding et al., 2009; Wang et al., 2010; Zhang et al., 2010). This ischaemia model has been shown to generate highly reproducible infarct volumes and cellular changes (Wang et al., 2010; Zhang et al., 2010). Using the PT model, we examined the effects of mGluR 1 antagonist, LY367385, and mGluR5 antagonist, MPEP, on acute and long-term brain damage, and the possible brain protective mechanism elicited by these antagonists.

## MATERIALS AND METHODS

### Animals

Male C57BL/6J mice aged 8–10 weeks were purchased from The Jackson Laboratory. All procedures were performed in accordance with the NIH (National Institutes of Health) Guide for the Care and Use of Laboratory Animals and were approved by the University of Missouri ACQA (Animal Care Quality Assurance) Committee.

### PT-induced brain ischaemia model

PT was induced similarly as described in our previous studies (Wang et al., 2010; Zhang et al., 2010). Briefly, mice were anaesthetized by ketamine and xylazine (130 mg/10 mg/kg body weight) and the photosensitive dye RB (rose Bengal) dissolved in saline was injected through the tail vein at a dose of 30 mg/kg. To induce PT, an area of 1.5 mm diameter in somatosensory cortex was focally illuminated for 2 min through a 10× objective with a green light of bandwidth 540–580 nm from an X-cite 120 PC metal halide lamp (EXFO). The power was set at 12% to activate the dye on the intact skull without skin at the centre of −0.8 mm from the bregma and 2.0 mm lateral to the midline. To study the effects of antagonists of group I mGluRs and calpain on ischaemia injury, MPEP (1 mg/kg), LY367385 (1 mg/kg), calpeptin (0.6 mg/kg) or their combinations were injected through tail vein 3 h after PT. The doses of the pharmacological reagents were determined based on literature and our previous studies (Makarewicz et al., 2006; Ding et al., 2007; Mani et al., 2008; Murotomi et al., 2008). Mice injected with saline were used as control. Mice were transcardially perfused at different times after PT for different studies. No mice died after ischaemia in our experiments since the infarct volume induced by PT was relative small as compared with that induced by MCAo.

### Transcardial perfusion and infarct volume and brain tissue loss measurements

The procedure was similar to our previous studies (Ding et al., 2009; Wang et al., 2010; Zhang et al., 2010). For histological studies, adult mice were transcardially perfused with PBS (pH7.4), followed by ice-cold 4% (v/v) PFA (paraformaldehyde) in PBS. After perfusion, the brain was removed and post-fixed in 4% PFA in PBS at 4°C overnight. It was then transferred to PBS with 30% (w/v) sucrose for 2–3 days until it sunk. Coronal sections of brain slices with a thickness of 30 μm were cut using a cryostat (CM1900, Leica) and were serially put on gelatin-coated glass slides or in a 48-well plate with 0.01 M PBS. To measure the infarct volume, every fifth brain slice on the glass slides was stained with 0.25% cresyl violet (Nissl-staining). The infarct volume was determined by measuring the areas showing the loss of Nissl-staining in brain sections (Schroeter et al., 2002; Vendrame et al., 2004; Wang et al., 2010; Zhang et al., 2010). The areas of cerebral infarction were delineated and quantified using the ImageJ software (NIH). The total infarct volume of ischaemic tissue was calculated by multiplying the individual infarct area by the total thickness of the five slices (150 μm). Brain tissue loss was estimated from the Nissl-stained brain sections in every fifth brain section (giving a distance of 150 μm between two sections). Tissue loss was quantified as the area of contralateral hemisphere minus the ipsilateral hemisphere and divided by the contralateral hemisphere. The areas of brain sections were calculated using the ImageJ software (NIH).

### FJB (fluoro-Jade B) and TUNEL (terminal deoxynucleotidyltransferase-mediated dUTP nick-end labelling) staining

For detecting degenerating neurons, brain sections were stained with FJB as described previously (Wang et al., 2010; Zhang et al., 2010). Briefly, brain sections fixed with 4% PFA on glass slides were washed with ddH_2_O (double-distilled water) and immersed in 0.06% (w/v) potassium permanganate for 20 min, and then immersed into 0.0004% FJB in 0.1% (v/v) acetic acid solution for 45 min in the dark. For detecting apoptotic neurons, TUNEL staining was performed using an *in situ* cell death detection kit (Roche) according to manufacturer's instructions. Briefly, brain tissue sections were incubated in freshly prepared permeabilization solution (0.1% (w/v) Triton X-100, 0.1% (w/v) sodium citrate) for 2 min on ice. TUNEL reaction mixture was then added on the brain sections and incubated in a humidified atmosphere at 37°C for 60 min in the dark. Images of the FJB- and TUNEL-stained sections were acquired with MetaMorph imaging software (Molecular Device) using a Nikon epi-fluorescence microscopy (Nikon) equipped with a CoolSNAP-EZ CCD (charge-coupled-device)-camera (Photometrics). The number of positive cells in the penumbra from each brain section was counted using the MetaMorph imaging software. Cell counting was performed by an experimenter blind to experimental conditions. Cells were counted if they contained a whole-cell body (Liu et al., 2009a; Zhang et al., 2010) and data were presented as number per mm^2^. The data from slices of each mouse brain were averaged and expressed as means±S.E.M.

### Immunostaining of brain slices

A floating method was used for immunofluorescent staining. Brain sections were immunostained with a mouse anti-GFAP (glial fibrillary acidic protein) mAb (monoclonal antibody) (1:800, Millipore), and a rat anti-BrdU (bromodeoxyuridine) mAb (1:200, Abcam). Brain sections were sequentially incubated with antibodies overnight at 4°C, then rhodamine-conjugated goat anti-mouse IgG (1:200, Millipore) or FITC-conjugated goat anti-rat IgG (1:200, Millipore) for 4 h at room temperature (21–23°C). Images were acquired in the same way as FJB- and TUNEL-stained slices.

### Western blot analysis

We used Western blot analysis to determine protein levels of calpain, GFAP and β-actin in the cortex. The procedures were similar to our previous studies (Zhang et al., 2010). Total protein was extracted from freshly harvested brain cortex using a lysis buffer plus protease inhibitor (Pierce Biotechnology) and phosphatase inhibitor cocktails (Sigma), pH 8.2. The homogenized tissue was centrifuged at 12000 ***g*** for 15 min at 4°C. The supernatant fluid is the total cell lysate. The protein concentration of the brain tissue lysate was determined with a BCA (bicinchoninic acid) protein assay kit (Pierce Biotechnology). Equivalent amounts of protein from each sample were diluted with Laemmli buffer, boiled for 5 min, subjected to SDS/PAGE (10% gels) at 100 mV and subsequently transferred to PVDF membranes. Membranes were blocked for 1 h with 5% (w/v) non-fat dried skimmed milk powder in TBST [Tris-buffered saline containing 0.1% (v/v) Tween 20] and were incubated overnight at 4°C in 3% (w/v) BSA with 0.02% (w/v) sodium azide in TBST with mouse anti-calpain antibodies (1:2000, sc-271313, Santa Cruz Biotechnology) or mouse anti-GFAP (1:2500, MAB360, Millipore) antibodies. The membranes were incubated with goat HRP (horseradish peroxidase)-conjugated anti-rabbit IgG (1:5000; Santa Cruz Biotechnology) diluted in 5% (w/v) non-fat dried skimmed milk powder in TBST for 1 h at room temperature. The membranes were then exposed to ECL) detection reagent (Pierce Biotechnology), and signals detected and scanned with a Fuji LAS 3000 densitometer. The ratios of integrated densities of the proteins (i.e. calpain and GFAP) to β-actin were calculated. Relative fold increases of calpain and GFAP were presented by normalizing the ratio of ipisilateral and contralateral sides of ischaemic mice to the control mice.

### BrdU injection and BrdU+ cell counting

We injected BrdU to label dividing cells in the penumbra and hippocampus. BrdU (Sigma) was administered daily through an intraperitoneal injection with a dose of 50 mg/kg in saline for 14 consecutive days starting from the same day after PT. Mice were killed 1 day after the last injection. The BrdU stock solution was 10 mg/ml in saline. For BrdU+ cell counting, brain coronal sections including DG (dentate gyrus) were selected for comparison using a mouse atlas. BrdU+ cells in the peri-infarct region and GCL (granular cell layer) in DG were counted. Counting of BrdU+ cells in GCL was made using 2–3 brain sections per mouse with each group of seven mice. Cells were counted if they contained a whole cell body. The data was presented as number per mm^2^ in the cortex or per DG region.

### Statistical analysis

Data are reported as means±S.E.M. Statistical comparisons were made by *t* test or a one-way ANOVA (Bonferroni *post hoc* test) for multiple groups. A *P*<0.05 was considered to be significant.

## RESULTS

Previously we used an open cranial window to induce ischaemia after RB injection (Wang et al., 2010; Zhang et al., 2010). In this study, we focally illuminated a green light in a region of 1.5 mm diameter on the exposed intact skulls in the somatosensory cortex for 2 min after RB injection. This protocol alleviated the surgical procedure for opening the cranial window. Blood flow measured using the laser Doppler flowmetry (Moor Instruments Inc.) dropped to 40% immediately after light illumination as compared with the basal level prior to illumination (Supplementary Figure S1; available at http://www.asnneuro.org/an/005/an005e117add.htm).

### Group I mGluR antagonists (MPEP and/or LY367385) reduce PT-induced ischaemic brain damage

**Figure 1:**
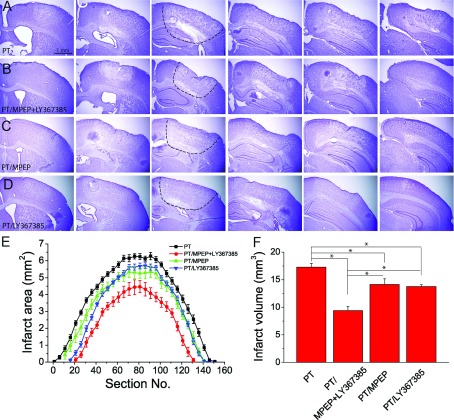
Effect of the inhibition of group I mGluRs on brain infarction after PT (**A**)–(**D**) Representative rostrocaudal series of Nissl-stained coronal sections showing the infarction regions of mice with the injections of saline (**A**), MPEP+LY367385 (**B**), MPEP (**C**) and LY367385 (**D**). Mice were killed 1 day after PT. The coronal brain sections have a thickness of 30 μm. The interval between brain sections is 750 μm (25 slices×30 μm). The dashed lines in third images in A–D) outline the infarct regions. (**E**) Infarct areas of rostrocaudal series of coronal brain sections from mice with different treatments. (**F**) Summary of brain infarct volumes. Data were average value of 7–21 mice for each group.*P<0.05, ANOVA test.

To test the effects of group I mGluRs on brain damage after PT, four group of mice were injected with saline (i.e. for the control group), the mGluR5 selective antagonist MPEP (1 mg/kg body weight), the mGluR1 selective antagonist LY367385 (1 mg/kg body weight) and the combination of the two antagonists (with the same doses) at 3 h after PT. At 24 h after PT, the mice were killed and brain sections were obtained for histological evaluation of ischaemia-induced tissue damage. Nissl-staining images showed that the infarcts were clearly demarcated from the surrounding region, indicating brain tissue damage. In addition, the underlying white matter was also partly affected by PT (Figures 1A–1D). Upon determination of infarct areas and volumes of each group (Figures 1E and 1F), the results show that a single injection of MPEP or LY367385 alone significantly reduced infarct area and volume as compared with the ischaemic group injected with saline (17.2 mm^3^ of control group versus 14.1 mm^3^ with MPEP injection and 13.5 mm^3^ with LY367385 injection; Figure 1F). In addition, co-injection of MPEP and LY367385 further reduced the infarct volume as compared with individual injections of MPEP or LY367385 (9.4 mm^3^ with co-injection of MPEP and LY367385; Figure 1F). These data suggest that the co-injection of MPEP and LY367385 has an addictive effect on the reduction of infarct volume in this condition. Since activation of mGluR1 and mGluR5 has the common PLC/IP_3_ pathway, the rest of the study will focus on the effect of co-injection of MPEP and LY367385 on ischaemic injury and its underlying mechanism, and the long-term histological outcomes.

### Inhibition of group I mGluRs by MPEP and LY367385 alleviates neuronal degeneration and apoptotic neuronal death after PT

**Figure 2:**
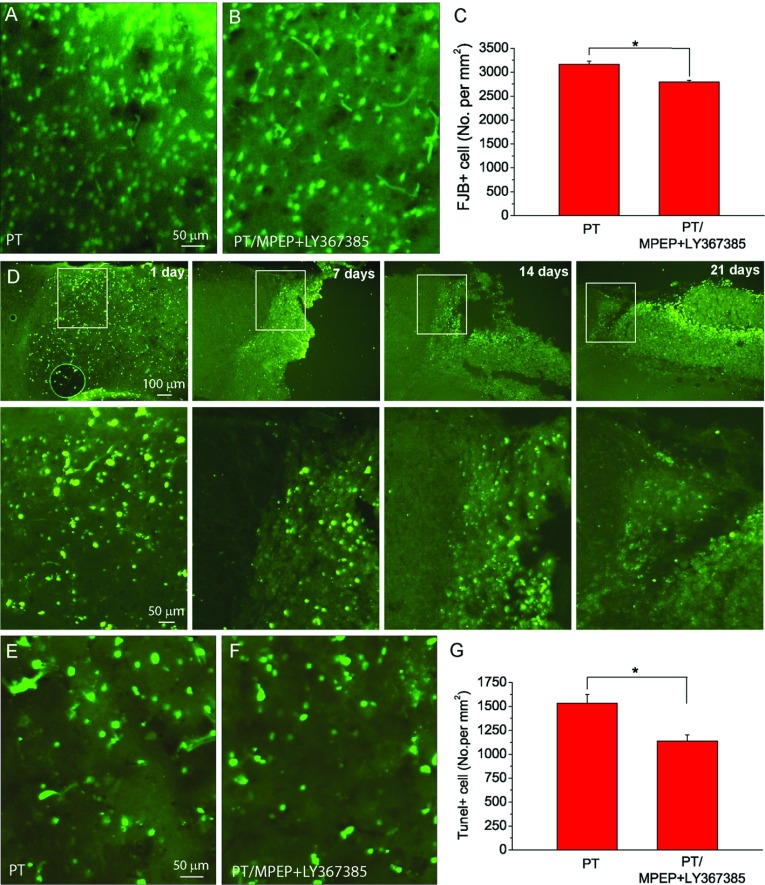
Inhibition of group I mGluRs by MPEP and LY367385 alleviates neuronal degeneration and apoptosis after PT (**A**,**B**) Representative images of FJB staining in the penumbra at 1 day after PT from mice injected with saline (**A**) and (**B**) MPEP and LY367385. (**C**) Summary of the densities of FJB+ cells after PT. The data were average values of three brain sections in the middle of infarction from each mouse (*N*=7 mice for the group with saline injection, and *N*=5 mice for the group with MPEP and LY367385 injection.*P<0.05, *t* test). (**D**) TUNEL staining images at different times as indicated after PT. Lower panels are the high resolution images of the boxed regions in the top panels. (**E**,**F**) Representative images of TUNEL staining in ischaemic region 1 day after ischaemia from mice injected with saline (**E**), and MPEP and LY367385 (**F**). (**G**) Summary of the densities of TUNEL+ cells in mice. The data were average values of two brain sections in the middle of infarction from each mouse (*N*=4 mice for the group with saline injection, and *N*=5 mice for the group with MPEP and LY367385 injection.*P<0.05, *t* test).

To further test the effects of MPEP and LY367385 on neurodegeneration in the penumbral regions, we performed FJB staining that specifically detects the degenerating neurons (Liu et al., 2009b; Wang et al., 2010; Zhang et al., 2010). By counting FJB+ cells in the penumbra, the results show that the density of FJB+ cell was slightly, but significantly lower in the MPEP and LY367385 co-injected mice as compared with the saline-injected mice (Figures 2A–2C).

Ischaemia induces not only necrotic cell death but also apoptotic cell death. To test the role of group I mGluRs in mediating apoptotic cell death, we used TUNEL staining to label apoptotic cells. Initially, we examined the time course of apoptosis following PT in mice without administration of antagonists. TUNEL assay was performed on brain sections from mice killed 1, 7, 14 and 21 days after PT. As shown in Figure 2(D), the density of apoptotic neurons in the penumbral region decreased gradually overtime up to 21 days with the highest TUNEL+ cell density at 1 day after PT. Therefore TUNEL staining was carried out at 1 day after PT to evaluate the effects of MPEP and LY367385 on apoptosis. Data show that the co-injection of MPEP and LY367385 significantly reduced the density of TUNEL+ cells in the penumbra as compared with the saline-injected mice (Figures 2E–2G). Taken together, the results from FJB and TUNEL staining indicate that group I mGluRs play an important role in neuronal degeneration and apoptosis after ischaemia.

### Group I mGluR antagonists suppress the increase in PT-induced calpain protein levels

**Figure 3:**
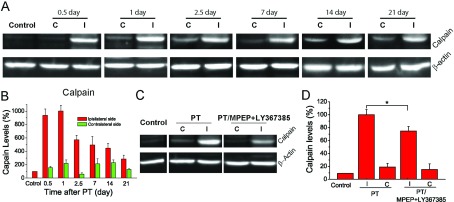
Co-administration of MPEP and LY367385 reduces the increased levels of calpain after PT (**A**) Western blot images of calpain expressions from the brain cortex at 0.5, 1, 2.5, 7, 14 and 21 days after PT. (**B**) Time course of calpain levels based on Western blot analysis after PT. β-actin was used as an internal control. Calpain expression levels were normalized to the level of mice with saline injection. *N*=4 mice for each time point. (**C**) Western blot images of calpain expressions from the brain cortex of mice injected with saline, and MPEP and LY367385 1 day after ischaemia. (**D**) The effect of MPEP and LY367385 injections on calpain after ischaemia. Calpain expression levels were normalized to the level of the ipsilateral side of mice with saline injections. *N*=8 mice for each group. *P<0.05, *t* test. C, contralateral site; I, ipsilateral side.

Calpain is a cytosolic Ca^2+^-activated protease and is known to be highly up-regulated after ischaemia (Bano et al., 2005; Vosler et al., 2011). Since MPEP and LY36738 can inhibit group I mGluRs-mediated signalling pathway and Ca^2+^ increase, it is conceivable that inhibition of group I mGluRs will reduce PT-mediated calpain expression. Western blot analysis showed that levels of calpain expression in the cortex of the ischaemic hemisphere were increased about 9-folds at 12 h after PT, and the increase was sustained for 24 h followed by a gradual decline over 21 days (Figures 3A and 3B). Interestingly, a small but significant increase in calpain expression was also observed in the contralateral side. Since calpain levels were highest 1 day after PT, we further tested the effects of co-administration of MPEP and LY367385 on calpain expression. Our data show that the PT-induced increase in calpain expression was suppressed to 74.8% by the co-administration of MPEP and LY367385 (Figures 3C and 3D). These data suggest that the protective effect of MPEP and LY367385 on the PT-mediated brain damage might be mediated, at least partly, through the suppression of calpain expression. It is necessary to emphasize that in this study, we can only measure the level of protein expression since calpain activity is difficult to measure with very small amount of ischaemic tissue.

**Figure 4:**
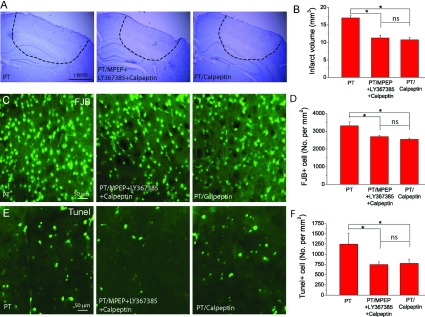
Effect of calpain inhibitor calpeptin on PT-induced brain infarction and neuronal death (**A**) Nissl stained images of brain sections from the regions with maximum infarction under different conditions, i.e. mice subject to PT (group 1, left), PT with administration of MPEP/LY367385/calpeptin (group 2, middle) and PT with administration of calpeptin (group 3, right). (**B**) Summary of infarct volumes. *N*=21 mice for group 1, *N*=4 mice for groups 2 and 3. (**C**,**D**) Representative images of FJB staining (**C**) and summary of the densities of FJB+ cell (**D**). (**E**,**F**) Representative images of TUNEL staining (**E**) and summary of the densities of TUNEL+ cell (**F**). *N*=4 mice for groups 1–3 in (**D**)–(**F**). *P<0.05, ns-no significant difference, ANOVA test.

To further investigate the mechanism and the involvement of calpain in MPEP and LY367385-mediated neuronal protection, we studied the effect of the calpain inhibitor, calpeptin on brain damage. Three groups of mice were used: (1) mice were subjected to PT; (2) mice were subjected to PT and co-injected with MPEP, LY367385 and calpeptin at 3 h after PT; and (3) mice were subjected to PT and injected with calpeptin alone at 3 h after PT. The results show that the group of mice injected with calpeptin alone produced similar infarct volumes with the group injected with MPEP, LY367385 and calpeptin (Figures 4A and 4B). Furthermore, the densities of FJB+ and TUNEL+ cells in groups 2 and 3 were similar but significantly lower than those in group I (Figures 4C–4F). These results suggest that inhibition of group I mGluRs by MPEP and LY367385 is sufficient to protect ischaemic damage through reducing calpain activation.

### Inhibition of group I mGluRs ameliorates infarction and tissue loss after long-term period of recovery from ischaemia

**Figure 5:**
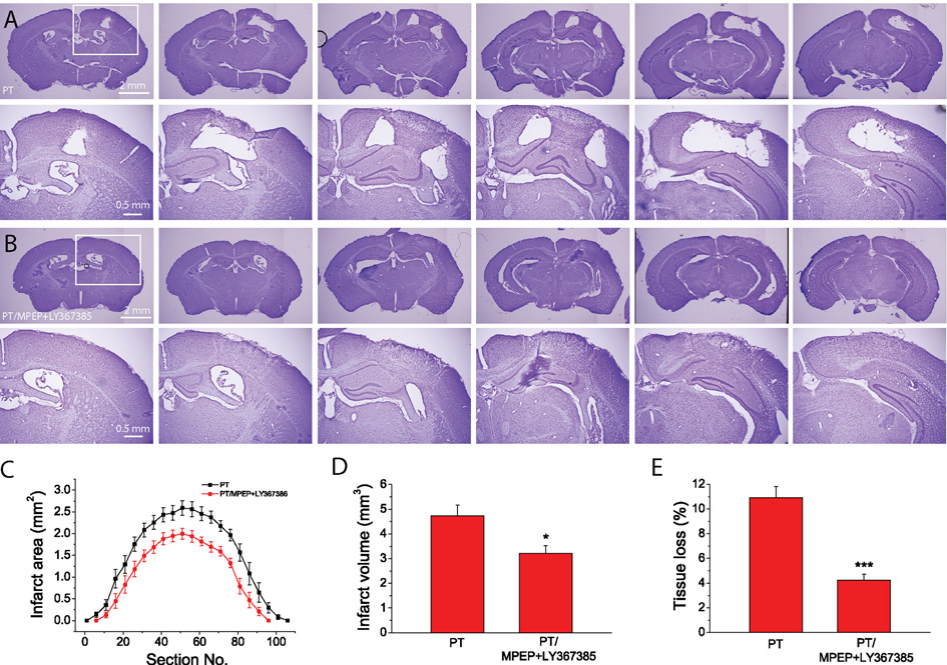
Long-term effect of MPEP and LY367385 on infarction and tissue loss after PT (**A**,**B**) Rostrocaudal series of Nissl-stained coronal sections showing the infarction 14 days after PT of mice without (**A**) and with (**B**) injection of MPEP and LY367385. Coronal brain sections were cut and displayed as in Figure 1. (**C**) Infarct areas of rostrocaudal series of brain sections. (**D**) Summary of brain infarct volumes. (**E**) Summary of tissue loss. Data in (**C**)–(**E**) were the average values of *N*=9 mice for each group. *P<0.05, ***P<0.0001, *t* test.

An important goal of brain protective study in ischaemia is to improve long-term outcomes. To this end, we evaluated the effects of MPEP and LY367385 on the infarct volume and tissue loss at 2 weeks after PT. Results show that co-injection of MPEP and LY367385 significantly reduced the infarct volume at this time point (3.2±0.3 mm^3^ versus 4.7±0.4 mm^3^; Figures 5A–5D). In addition, we observe that mice not subjected to antagonist administration had noticeable holes near the infarction, presumably due to the shrinkage of surrounding tissue (Figure 5A), whereas mice subjected to antagonists administration exhibited compact ischaemic tissue (Figure 5B). Brain tissue loss as analysed by Nissl staining was significantly reduced in mice co-injected with MPEP and LY367385 as compared with mice injected with saline (4.2±0.5% versus 10.9±0.9%; Figure 5E). These data demonstrate that MPEP and LY367385 can significantly protect brain from long-term damage.

### Inhibition of group I mGluRs perturbs glial scar formation and astrocyte reactivity in the penumbral region

**Figure 6:**
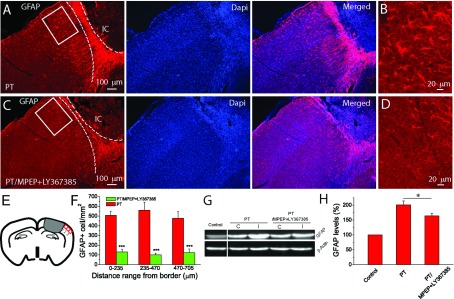
MPEP and LY367385 attenuate glial scar formation and GFAP levels in the ipsilateral side of brain 2 weeks after PT (**A**)–(**D**) GFAP staining images at 2 weeks after PT without (**A**,**B**) and with (**C**,**D**) MPEP and LY367385 injection. (**B**) and (**D**) are the high-resolution images of the boxed region in (**A**) and (**C**). The dashed lines in (**A**) and (**C**) indicate the boundary of glial scar. IC, ischaemic core. (**E**) Illustration of the regions for the counting of GFAP+ reactive astrocyte. (**F**) The density of GFAP+ reactive astrocytes at different distances from the ischaemic border in mice with and without injection of MPEP and LY367385. The data were average values of two brain sections in the middle of infarction from each mouse (*N*=4 mice for the group without drug injection, and *N*=5 mice for the group with MPEP and LY367385 injection).*P<0.0001, *t* test. (**G**) Western blot image of GFAP expression levels in the cortex in the ipsilateral side of mice 2 weeks after PT. (**H**) Summary of GFAP expression levels in the cortices determined from Western blot. Notice the significant reduction of GFAP levels in the mice injected with MPEP and LY367385. The GFAP levels were normalized to the non-ischaemic mice and data were averaged from *N*=5 mice for each group. *P<0.05, *t* test.

Glial scar formation is a major event in the recovery process after ischaemia and up-regulation of GFAP expression in astrocytes is the hallmark of glial scar and astrogliosis. Since it is known that group I mGluRs are associated with Ca^2+^ signalling pathway in astrocytes (Zonta et al., 2003; Wang et al., 2006; Ding et al., 2007, 2009), it is possible that the glia scar formation is altered with the administration of the antagonists of group I mGluRs. In mice injected with saline, immunostaining of brain sections 14 days after PT showed strong signals of GFAP in the surrounding area of the ischaemic core, and high density of GFAP+ cells appeared even in the regions distant from the ischaemic core. In contrast, mice injected with mGluR antagonists had less GFAP+ cells surrounding the ischaemic core (Figures 6A–6D) and thinner glial scar than mice injected with saline (the regions between the dashed lines in Figures 6A and 6C). Quantitative analysis indicates that astrocyte density in the ischaemic border is also lower in antagonists-injected mice as compared with saline-injected mice (Figure 6F). Western blot analysis further confirmed the reduction in levels of GFAP expression in the cortices of the ipsilateral hemisphere after administration of MPEP and LY367385 (163.8±8.5% versus 201.0±13.0%; Figures 6G and 6H).

### Inhibition of group I mGluRs reduces cell proliferation in the penumbra

**Figure 7:**
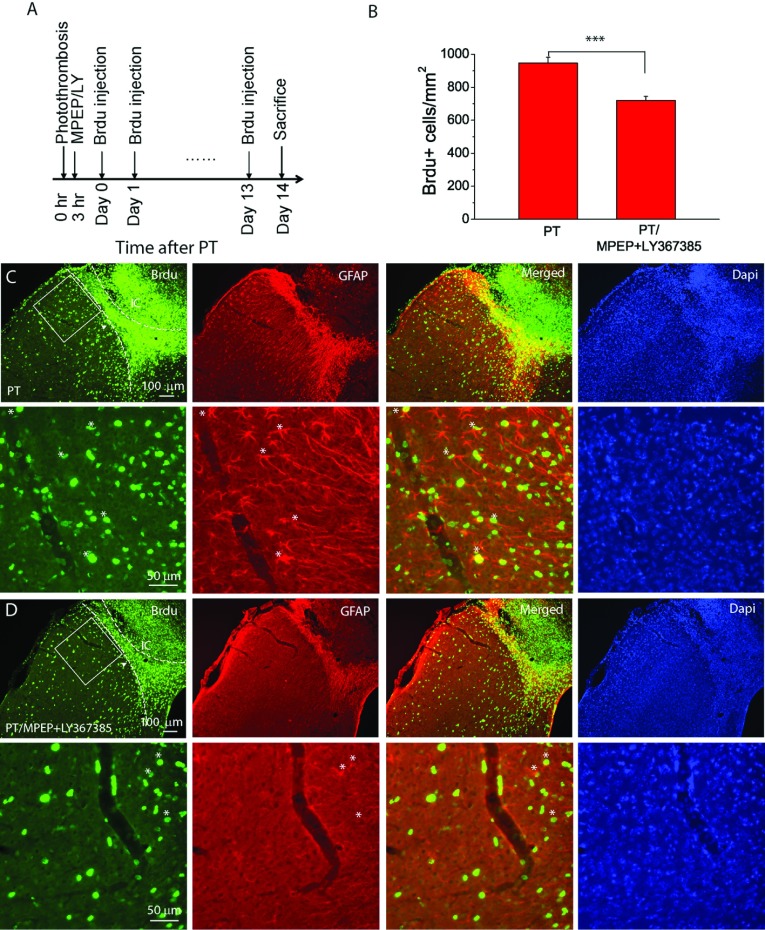
MPEP and LY367385 attenuate cell proliferation in the peri-infarct region after PT (**A**) Experimental design of BrdU injections for cell proliferation study. BrdU+ was injected in mice once a day starting the same day after PT for 14 times and mice were killed 1 day after last injection. (**B**) Summary of the density of BrdU+ cell in a peri-infarction region of 325 μm×325 μm in the layer 2/3 of cortices (see boxed region in (**C**,**D**). The data were averaged from *N*=7 mice for each group. *P<0.0001, *t* test. (**C**,**D**) Fluorescent images of BrdU and GFAP staining in the infarct and peri-infarct regions of mice not subject (**C**) or subject (**D**) to MPEP and LY367385 injection. The left-hand dashed lines indicate boundary of dense BrdU+ cell, and the right-hand dashed lines indicate the boundary of proliferating cells in glial scars based on GFAP staining. IC, ischaemic core. The bottom panels in (**C**) and (**D**) are the high-resolution images of the boxed regions in the upper panels and some of the BrdU+/GFAP+ cells are indicated by *. Note there are more BrdU+/GFAP+ cells in (**C**) than in (**D**).

Glia scar formation is known to associate with proliferation of cells including reactive astrocytes in the penumbra. To examine the effect of MPEP and LY367385 on cell proliferation, we injected BrdU once daily for 14 days starting from the day of PT (Figure 7A). Dense BrdU+ cells were observed in the penumbral region surrounding the ischaemic core (see the regions between the two dashed lines in Figures 7C and 7D). The density of BrdU+ cells in the region adjacent to the penumbra in mice injected with MPEP and LY367385 was attenuated as compared with that of mice injected with saline. Quantitatively analysis of the region with a size of 325 μm×325 μm in the layer 2/3 from the boundary of the penumbra (boxed regions in Figures 7C and 7D) showed a significant reduction of BrdU+ cell density in mice injected with MPEP and LY367385 (720 cells/mm^2^ versus 946 cells/mm^2^; Figure 7B). In addition, double staining of BrdU and GFAP indicates more BrdU+/GFAP+ cells (indicated with * in Figures 7C and 7D) in mice injected with saline as compared with those injected with the antagonists. Thus, our results demonstrate that inhibition of group I mGluRs also lead to attenuation of cell proliferation and glial scar formation after ischaemia.

Since it is well documented that ischaemia increases neurogenesis in DG in the hippocampus (Zhu et al., 2003; Ohab et al., 2006; Wang et al., 2009), we also counted BrdU+ cell in the SGZ (subgranular zone) of DG (Supplementary Figure S2; available at http://www.asnneuro.org/an/005/an005e117add.htm). Our results showed that although the number of BrdU+ cell was higher in the SGZ of the ipsilateral side as compared with the contralateral side, administration of MPEP and LY367385 did not affect the density of BrdU+ cell in the SGZ in both brain hemispheres. Thus, the effect of MPEP and LY367385 on cell proliferation is limited to the penumbral regions of the ischaemic core.

## DISCUSSION

Using the PT-induced ischaemia model in adult mice, we observed the following findings in the present study: (1) A single injection of the mGluR5 antagonist, MPEP or the mGluR1 antagonist, LY367385, could produce brain protective effects in ischaemia, and their co-injection with the same dosage further reduced the infarct volume. Data from FJB and TUNEL staining confirmed the effects of MPEP and LY367385 on reduction of neuronal degeneration and apoptotic cell death. (2) A single co-injection of MPEP and LY367385 could exert a long-term neuroprotective effect by reducing infarction and tissue loss evaluated 2 weeks after PT. (3) Based on the results of Western blot analysis, co-injection of MPEP and LY367385 effectively reduced PT-induced Ca^2+^-activated protease calpain expression in the ischaemic hemisphere. Calpain inhibitor calpeptin alone reduced the infarction and neuronal death after PT but injection of calpeptin together with MPEP and LY367385 did not further improve protective effects as compared with the injection of the antagonists. These results suggest that the brain protective effect mediated by MPEP and LY367385 is at least partially through the suppression of calpain. (4) Co-injection of MPEP and LY367385 could attenuate glial scar formation and cell proliferation in the peri-infarct region at 2 weeks after ischaemia. Taken together, this study demonstrated the involvement of group I mGluRs in short- and long-term ischaemic brain injury and protective effects of the antagonists.

Glutamate is the major excitatory neurotransmitter in the CNS. The vast amount of glutamate release and the subsequent Ca^2+^ overloading after brain injury, including ischaemia, is excitotoxic. Glutamate excitotoxicity is the primary cause of acute neuronal death (necrosis) and initiates apoptosis after ischaemia (Dirnagl et al., 1999). Although many studies have investigated the role of group I mGluRs in ischaemia, the underlying basic mechanisms have not been well understood. Studies have shown the correlation of neuroprotective effects of mGluR1 antagonist to the decrease in tyrosine phosphorylation of NMDAR, NADPH oxidase activity and superoxide production after MCAo in mice (Murotomi et al., 2008, 2010). It has also been shown that selective mGluR1 antagonists are neuroprotective through enhancing GABA (γ-aminobutyric acid) release (Battaglia et al., 2001; Cozzi et al., 2002). It is known that stimulation of group I mGluRs, including mGluR1 and mGluR5, increases intracellular Ca^2+^ levels, and calpain is a Ca^2+^-activated protease that is up-regulated after ischaemia. Activation of calpain leads to neuronal death through degrading different proteins (Goll et al., 2003), and its inhibition is neuroprotective in ischaemia (Bano et al., 2005; Cao et al., 2007; Vosler et al., 2011). Therefore we investigated the involvement of calpain in MPEP and LY367385-mediated neuroprotection in ischaemia. Our data show that injection of calpain inhibitor, calpeptin, could reduce infarct volume and neuronal death similar to the co-injection of MPEP and LY367385. However, co-injection of calpeptin with MPEP and LY367385 did not further reduce infarct volume and neuronal death as compared with co-injection with MPEP and LY367385, and with the injection of calpeptin alone. These results suggest that inhibition of group I mGluRs by MPEP and LY367385, is sufficient to protect ischaemic damage through reducing calpain activity possibly via the suppression of Ca^2+^ release. Although many studies have related to a neuronal source of calpain, it is not possible to differentiate the sources of calpain in this study. Nevertheless, we did not observe a colocalization of calpain with the astrocyte-specific markers GFAP and S100B at 1 day after PT using immunostaining (results not shown). To our knowledge there was no report on calpain up-regulation in astrocyte *in vivo* after ischaemia, although it is still possible that astrocytes can mediate calpain increase in the brain in an indirect way.

Neuronal Ca^2+^ increase is largely due to the activation of ionotropic glutamate receptors including NMDARs and AMPARs whereas GPCRs including group I mGluRs-mediated Ca^2+^ increase is known as the main mechanism of astrocytic excitability. Our recent study showed that astrocytes mediate enhanced Ca^2+^ signalling after PT-induced ischaemia (Ding et al., 2009), and the ischaemia-induced Ca^2+^ signals in astrocytes can be largely inhibited by mGluR5 antagonist MPEP. It is possible that the increase in Ca^2+^ in astrocytes can in turn induce glutamate release and contribute to ischaemia-induced glutamate excitotoxicity. Our previous study from brain slice recording and *in vivo* imaging also showed that MPEP could reduce neuronal excitation and inhibit CHPG/DHPG-stimulated Ca^2+^ increase in astrocytes (Ding et al., 2007). A recent study showed that astrocytic mGluR5 is developmentally regulated with lower expression levels in adult mice than in neonatal mice (Sun et al., 2013); however, it is also shown that mGluR5 is up-regulated in astrocytes after oxygen glucose deprivation (Paquet et al., 2013). Thus, it remains to test whether the brain protective effect of MPEP and LY367385 in ischaemia is associated with their actions on astrocytes. Given that both neurons and astrocytes express group I mGluRs, it is necessary to use astrocyte-specific mGluR1 or mGluR5 knockout mice to unequivocally determine the role of astrocytic group I mGluRs in brain protective effects after ischaemia.

We also studied the long-term effects of MPEP and LY367385 on brain recovery after ischaemia. A single injection at 3 h after PT significantly reduced infarct volume and tissue loss evaluated 2 weeks later suggesting that inhibition of group I mGluRs can elicit a long-lasting effect on post-ischaemia recovery. In focal cerebral ischaemia, it is a common phenomenon to observe glial scars comprised with reactive astrocytes with highly up-regulated GFAP expression levels. Our results indicate that administration of MPEP and LY367385 reduced glial scar thickness and the density of GFAP expressing astrocytes in the peri-infarct regions. In addition, the overall GFAP levels in the cortex of the ipsilateral hemisphere in mice injected with MPEP and LY367385 were significantly lower than those in mice injected with saline. Using the BrdU+ labelling method, we found that MPEP and LY367385 significantly attenuated cell proliferation in the region near the penumbra. It is reported that double knockout mice of GFAP and vimentin have larger infarction than WT (wild-type) mice after ischaemia (Li et al., 2007) and improve axonal plasticity and functional recovery after spinal cord injury (Menet et al., 2003). Ablation of reactive astrocytes can also exacerbate spinal cord injury (Faulkner et al., 2004; Sofroniew, 2005). These studies suggest that reactive astrocytes play a protective role in ischaemia and spinal cord injury. On the other hand, reactive astrocytes are implicated as inhibitors of neuroregeneration in the entorhinal cortex lesion model (Wilhelmsson et al., 2004). In ischaemia, attenuation of glial scar formation is associated with either increase or decrease in infarct volumes (Bao et al., 2012; Shen et al., 2012). Although we cannot draw a conclusion as to whether glial scar has beneficial or detrimental effects on neuronal and brain protection after ischaemia, our results using the PT model show that inhibition of group I mGluRs with MPEP and LY367385 is associated with attenuation of glial scar formation and cell proliferation. This reduction could be a secondary effect to a decrease in injury mediated by the antagonists or could represent a direct effect on astrocyte dynamics.

In summary, the present study provides evidence that group I mGluRs play an important role in neuronal death and damage in adult mice after ischaemia. The protective effects of the antagonists are associated with the suppression of ischaemia-induced increase in Ca^2+^-activated calpain. In addition, inhibition of group I mGluRs can also improve long-term histological stroke outcomes and attenuate glial scars and cell proliferation in the penumbra. However, further studies with cell-type-specific knockout mice are needed to distinguish the role of neuronal and astrocytic group I mGluRs in ischaemia.

## Online data

Supplementary data
