# Supplementary material for: Inhibition of the group I mGluRs reduces acute brain damage and improves long-term histological outcomes after photothrombosis-induced ischaemia
Source: ASN Neuro. 2013 Jul 11;5(3):e00117. doi: 10.1042/AN20130002 (PMC3786425; doi:10.1042/AN20130002)
Supplement: Supplementary data [file an005e117add.pdf]

ASN NEURO RESEARCH ARTICLE

ASN NEURO 5(3):art:e00117.doi:10.1042/AN20130002

OPEN ACCESS

Inhibition of the group I mGluRs reduces acute brain damage and improves long-term histological outcomes after photothrombosis-induced ischaemia

Hailong Li*†, Nannan Zhang*, Grace Sun‡ and Shinghua Ding†[1]

*Dalton Cardiovascular Research Center, University of Missouri-Columbia, MO 65211, U.S.A.

†Department of Biological Engineering, University of Missouri-Columbia, MO 65211, U.S.A.

‡Department of Biochemistry, University of Missouri-Columbia, MO 65211, U.S.A.

SUPPLEMENTARY DATA

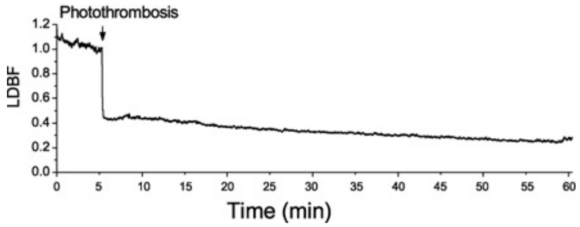

Figure S1 Cerebral blood flow (CBF) measurement of photothrombosis model with a laser Doppler flowmetry
In this experiment, photothrombosis was induced by 2 min of light illumination of 12 % power output of light source on a region with a diameter of 1.5 mm in the intact skull without skin. CBF was monitored up to 1 h after photothrombosis. Data from each mouse was normalized to the value prior to light illumination. The Figure shows the averaged value of data from five mice.

[1]To whom correspondence should be addressed (email dings@missouri.edu).

© 2013 The Author(s) This is an Open Access article distributed under the terms of the Creative Commons Attribution Licence (CC-BY) (http://creativecommons.org/licenses/by/3.0/) which permits unrestricted use, distribution and reproduction in any medium, provided the original work is properly cited.

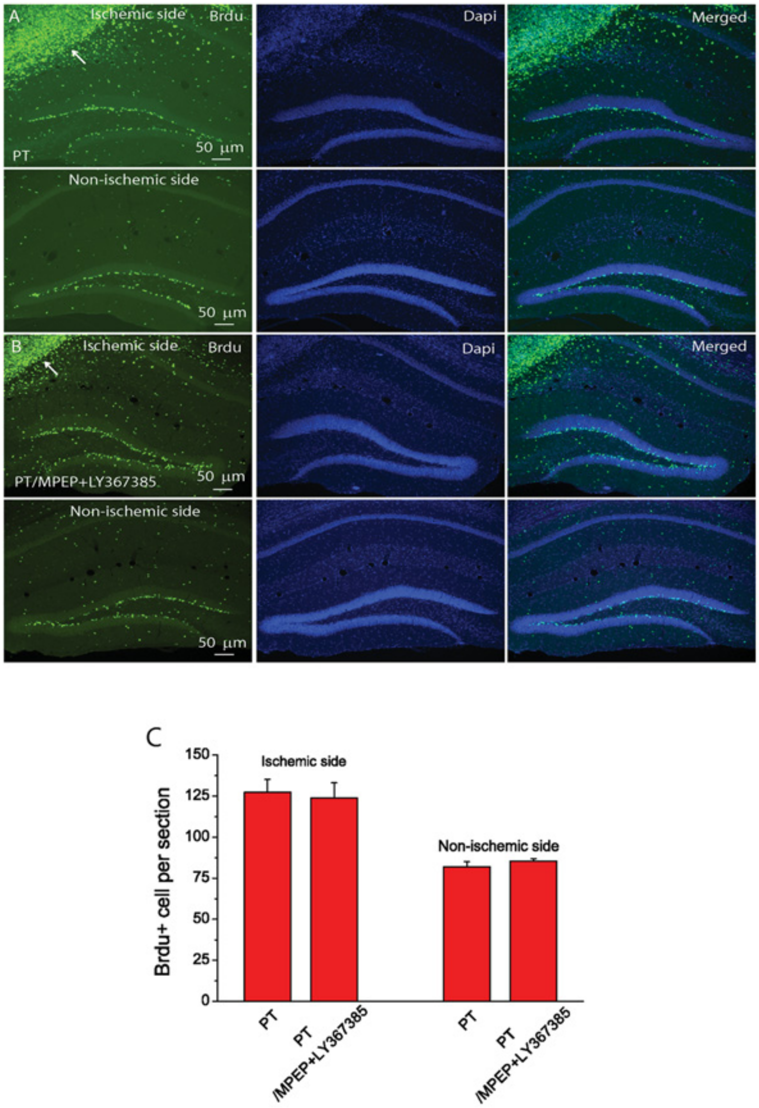

Figure S2 **The effect of MPEP and LY367385 on neurogenesis in the hippocampus 2 weeks after PT**
(**A**,**B**) Fluorescent images of Brdu staining in the dentate gyrus of hippocampus of mice not subject (**A**) and subject (**B**) to MPEP and LY367385 injection. (**C**) Summary of the density of Brdu + cells in the SGZ of DG. The Brdu injection paradigm is the same as in Figure 6(A) of the main text.

© 2013 The Author(s) This is an Open Access article distributed under the terms of the Creative Commons Attribution Licence (CC-BY) (http://creativecommons.org/licenses/by/3.0/) which permits unrestricted use, distribution and reproduction in any medium, provided the original work is properly cited.
